# Supplementary material for: Chronic Binge Alcohol and Ovarian Hormone Loss Dysregulate Circulating Immune Cell SIV Co-Receptor Expression and Mitochondrial Homeostasis in SIV-Infected Rhesus Macaques
Source: Biomolecules. 2022 Jul 5;12(7):946. doi: 10.3390/biom12070946 (PMC9313096; doi:10.3390/biom12070946)
Supplement: Supplementary file 1 [file biomolecules-12-00946-s001.zip › Supplemental Files/Supplemental_Figure_S3.pptx]

## Slide 1
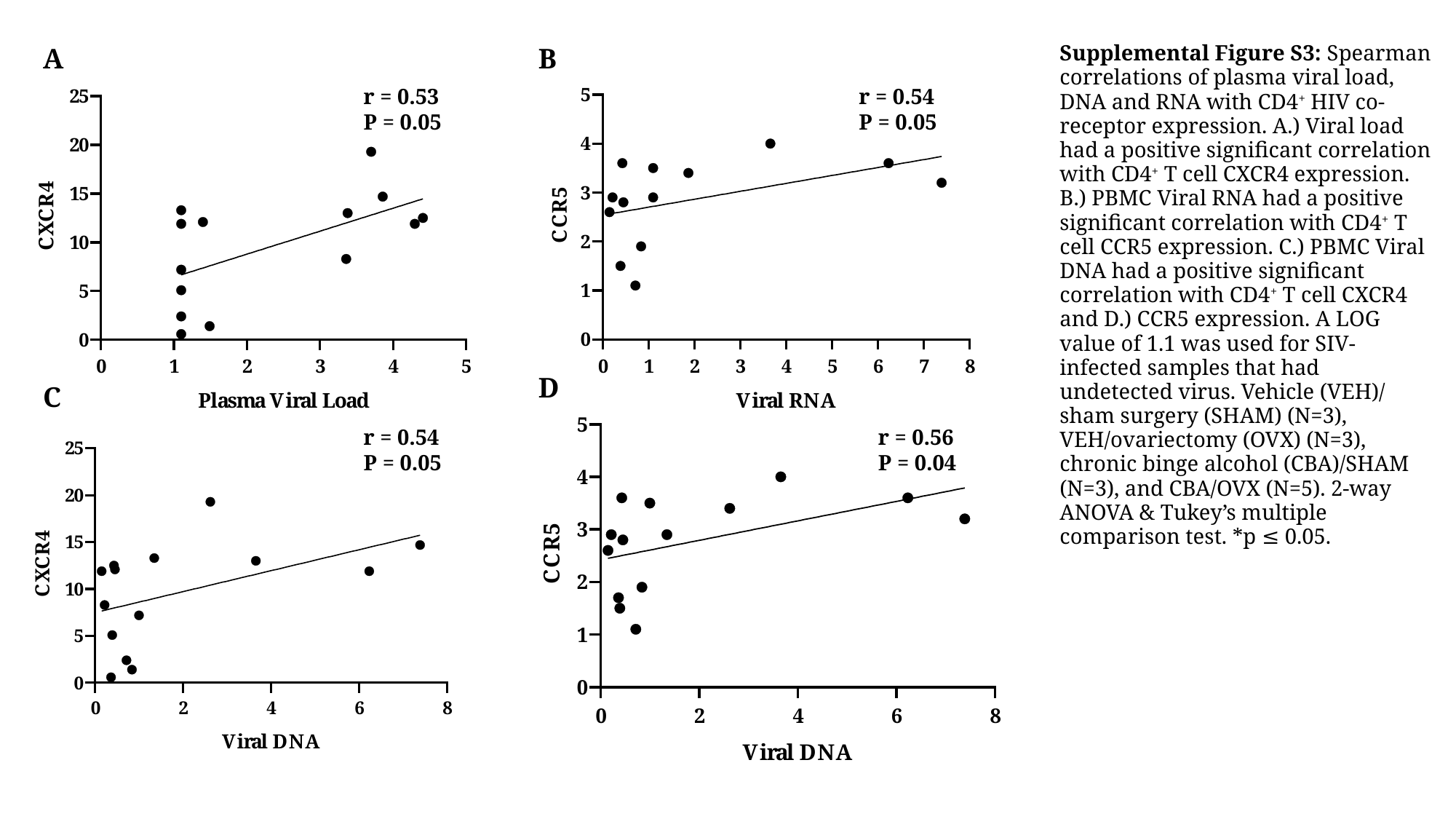

A
B
r = 0.54
P = 0.05
r = 0.53
P = 0.05
D
C
r = 0.56
P = 0.04
r = 0.54
P = 0.05
Supplemental Figure S3: Spearman correlations of plasma viral load, DNA and RNA with CD4+ HIV co-receptor expression. A.) Viral load had a positive significant correlation with CD4+ T cell CXCR4 expression. B.) PBMC Viral RNA had a positive significant correlation with CD4+ T cell CCR5 expression. C.) PBMC Viral DNA had a positive significant correlation with CD4+ T cell CXCR4 and D.) CCR5 expression. A LOG value of 1.1 was used for SIV-infected samples that had undetected virus. Vehicle (VEH)/ sham surgery (SHAM) (N=3), VEH/ovariectomy (OVX) (N=3), chronic binge alcohol (CBA)/SHAM (N=3), and CBA/OVX (N=5). 2-way ANOVA & Tukey’s multiple comparison test. *p ≤ 0.05.
