# Supplementary material for: Chronic Binge Alcohol and Ovarian Hormone Loss Dysregulate Circulating Immune Cell SIV Co-Receptor Expression and Mitochondrial Homeostasis in SIV-Infected Rhesus Macaques
Source: Biomolecules. 2022 Jul 5;12(7):946. doi: 10.3390/biom12070946 (PMC9313096; doi:10.3390/biom12070946)
Supplement: Supplementary file 1 [file biomolecules-12-00946-s001.zip › Supplemental Files/Supplemental_table_S1.pptx]

## Slide 1
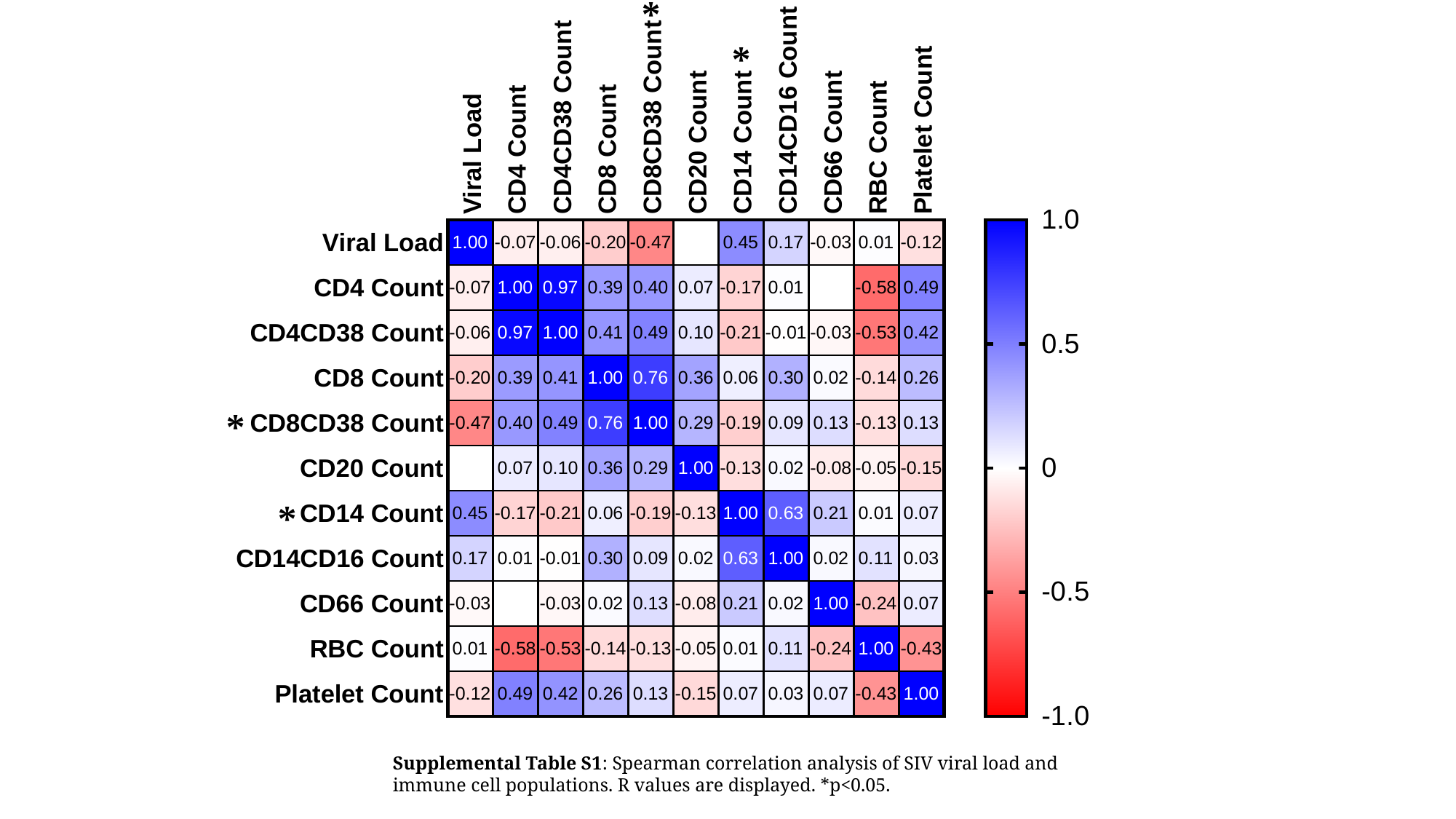

*
*
*
*
Supplemental Table S1: Spearman correlation analysis of SIV viral load and immune cell populations. R values are displayed. *p<0.05.
