# Supplementary figures and images for: Chronic Binge Alcohol and Ovarian Hormone Loss Dysregulate Circulating Immune Cell SIV Co-Receptor Expression and Mitochondrial Homeostasis in SIV-Infected Rhesus Macaques
Source: Biomolecules. 2022 Jul 5;12(7):946. doi: 10.3390/biom12070946 (PMC9313096; doi:10.3390/biom12070946)

## Slide 1
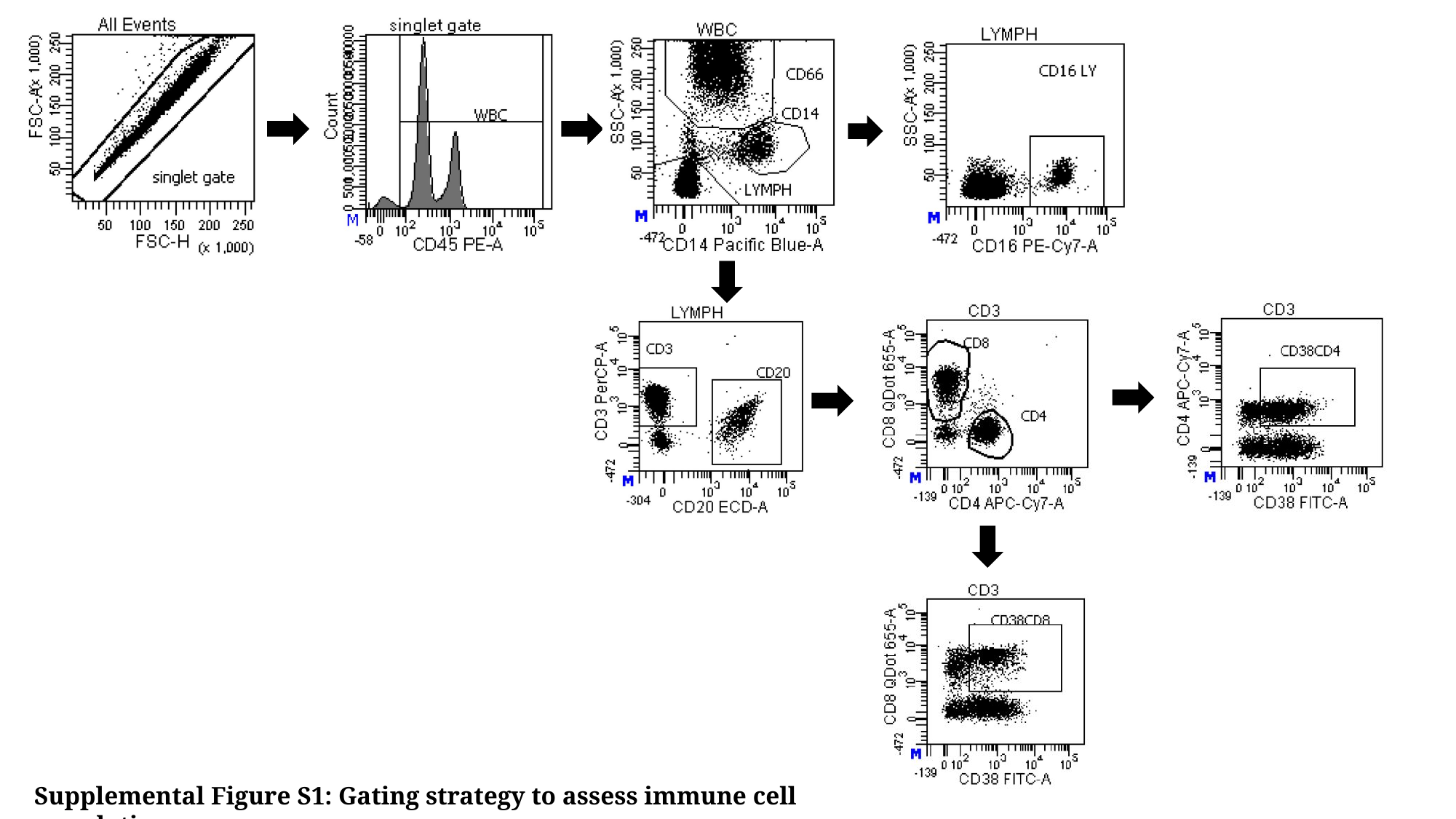

Supplemental Figure S1: Gating strategy to assess immune cell populations.

Supplement: Supplementary file 1 [file biomolecules-12-00946-s001.zip › Supplemental Files/Supplemental_Figure_S1.pptx]

## Slide 1
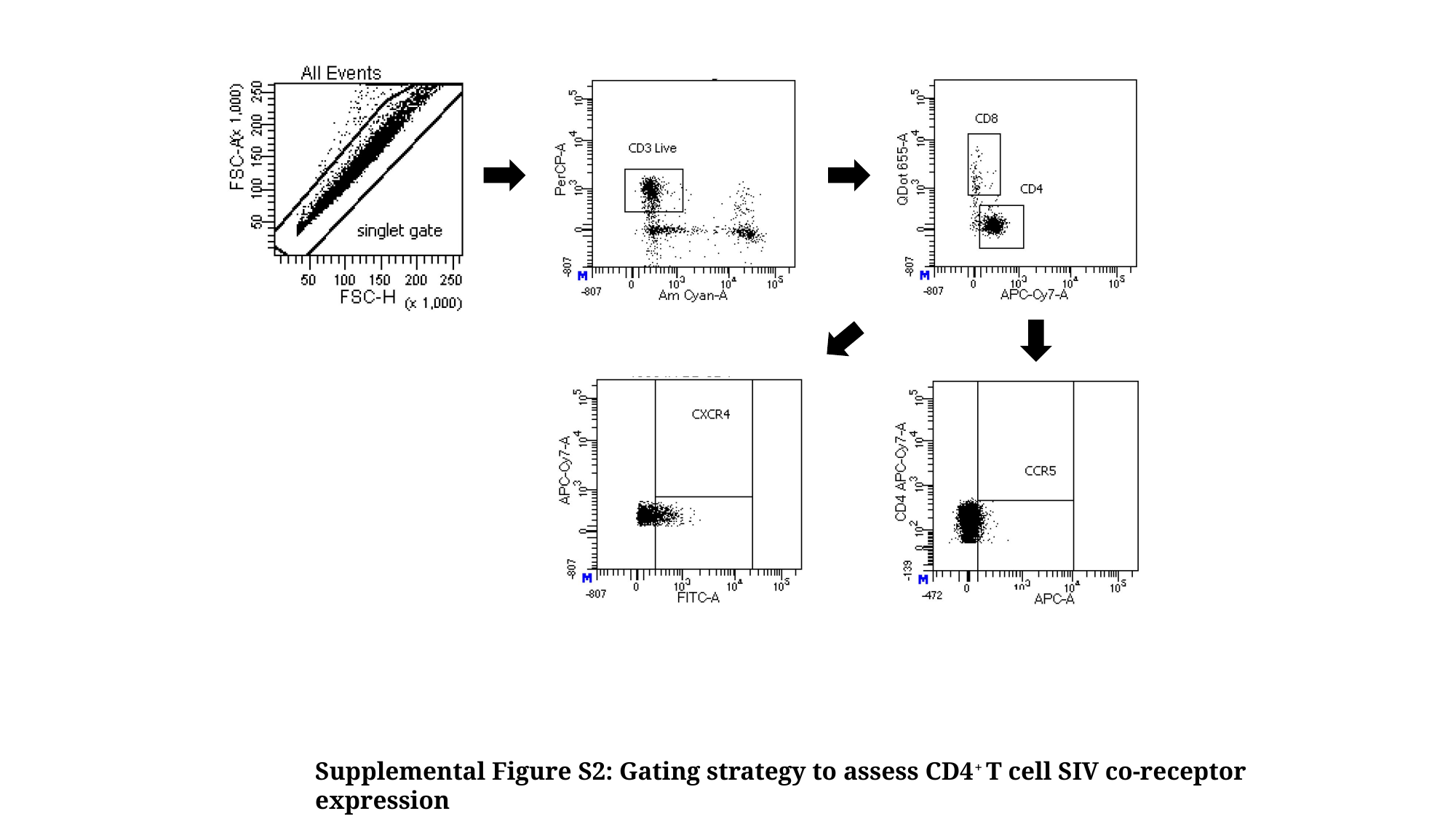

Supplemental Figure S2: Gating strategy to assess CD4+ T cell SIV co-receptor expression

Supplement: Supplementary file 1 [file biomolecules-12-00946-s001.zip › Supplemental Files/Supplemental_Figure_S2.pptx]
